# Maintaining US Leadership in Emerging Biotechnologies to Grow the Economy of the Future

**DOI:** 10.1089/hs.2016.0099

**Published:** 2017-02-01

**Authors:** Gigi Kwik Gronvall

According to the US National Bioeconomy Blueprint (2012), applications emerging from synthetic biology and other biotechnologies “can allow Americans to live longer, healthier lives, reduce our dependence on oil, address environmental challenges, transform manufacturing processes, and increase the productivity and scope of the agricultural sector while growing new jobs and industries.”^1^^(p1)^ In addition to benefits to these diverse aspects of American life, biotechnology's potential effect on growing the economy is thought to be immense. Synthetic biology, a technical area that aims to make biology more useful and easier to engineer, is a top 10 key technology for the 21st century, according to the World Economic Forum. BCC Research, a market analysis company, expects the synthetic biology market to grow rapidly from $2.7 billion in 2013 to $11.8 billion in 2018, with a compound annual growth rate of 34.4% over a 5-year period from 2013 to 2018.

Other nations have taken note of the potential for biotechnology to fuel their economic development and have invested accordingly, particularly the UK and China. At the same time that these technologies are seen as a lucrative area for investment by nations, there are increasing concerns that the United States is falling behind in science generally, and that the STEM (science, technology, engineering, and mathematics) workforce is declining. The National Institutes of Health has warned of an “erosion of the competitive position of the US life sciences industry over the past decade.” China will overtake the United States in R&D spending by 2020 and has already exceeded the number of doctoral degrees awarded in the life sciences in the United States. The US is currently the leader in synthetic biology and other biotechnologies—but for how long this will remain the case is in question.

A forewarning of the US biotechnology future can be seen in the iGEM (internationally genetically engineered machines) synthetic biology competition. This international competition, which is similar in scope to better-known robotics competitions, started as a class at MIT in 2003. By 2016, it had grown into a premier competition for synthetic biology, with 5,200 participants, spanning 42 countries. It is notable that none of this year's trophy winners hailed from the United States: The winners came from the UK, Australia, China, the Netherlands, Germany, and Taiwan. According to a committee assembled by MIT, the US teams have suffered for years from a lack of laboratory and institutional support.

This trend of decline must be reversed. If the United States is not a leader in the newest areas of biotechnology like synthetic biology, it will lose out on the economic, biomedical, agricultural, and manufacturing benefits that are widely expected to ensue. Given the potential that these technologies could also be used for harm, it would also lose out on opportunities to shape the field as well as to use it for the nation's defense.

## Recommendations

➢ **The US should remain competitive in S&T, including in synthetic biology.**

Maintaining competitiveness in science and technology (S&T), including synthetic biology, involves following a well-known playbook to which the US government should adhere: The United States should fund basic research with minimal fluctuations from year to year; fund STEM education initiatives; institute financial incentives to discourage synthetic biology companies and other biotechnology companies from locating offshore; develop the workforce through training programs; ensure that women are targeted for advancement in STEM fields; and encourage foreign students who receive their PhDs in the United States in technical areas to stay in the country, by issuing green cards enabling them to work here. Targeted initiatives to make the United States an innovative space for science and scientific research generally will help the United States maintain its leadership position across the S&T spectrum, including in synthetic biology. Specific to US leadership in synthetic biology, the nation should invest in the scientists of the future and give more support to university iGEM teams to help them succeed.

➢ **The United States should continue to support a regulatory philosophy that focuses on the end product, which has proven beneficial to innovation, and not a “precautionary” European model. Although there is mounting sentiment against genetically modified organisms (GMO) in the United States, which is counterproductive to US excellence in biotechnology, the regulatory system for biotechnology in this country should continue to be based on sound science, and should not be politically driven.**

The US approach to regulating biotechnology differs from that typically embraced in Europe. In the United States, it is the *product* that needs to be subject to a safety determination, not the process used to *make* a product. This encourages innovation, to come up with better methods to reach the same desired result. European regulatory systems generally adhere to the “precautionary principle,” which places the burden of proof on the developer of a product to show that the process used to make a product is not harmful. While precaution in the face of unknown risks sounds like a reasonable approach—it is the essence of the expression “look before you leap”—in practice, it has often resulted in inaction and has discouraged innovation.

The divergence of a precautionary and more typically American pro-actionary regulatory philosophy is evident in the debate about GMOs. In the United States, whether a product is made through GMOs or another method, it is the end-product that is regulated. In Europe, the act of genetically modifying organisms is severely restricted and under tight regulatory control. As synthetic biology has been termed “genetic engineering on steroids,” the current US regulatory approach is more favorable to synthetic biology development and should be maintained even if there is pressure from anti-GMO activists. The United States is in the midst of updating its Coordinated Framework for Regulating Biotechnology Products—that is, the division of responsibilities among the Environmental Protection Agency, the US Department of Agriculture (USDA), and the FDA, which all have oversight responsibilities for biotechnology products—and should maintain a system that is based on science.

➢ **The United States should ensure that products made in synthetic biology are appropriately regulated and overseen.**

The US regulatory system for biotechnology products should be driven by science and, importantly, should not have gaps that may sow public distrust in the regulatory oversight. The US regulatory system needs to be well-equipped to assess the risks associated with future biotechnology products, particularly as synthetic biology is likely to increase the gaps in regulation. As one example, a 2013 fundraising campaign on Kickstarter alarmed experts by promising to produce glowing plants and distributing their seeds for planting (in the end, the technical obstacles were too great, and the company was not able to deliver this product). The activities were not forbidden under any regulation, though many reasonably thought this would be an activity that should be subject to regulation.

The regulatory framework is being updated, and this process should continue through the next Administration. The last time the framework was updated was in 1992, a very long time ago in terms of biotechnology development. An outdated regulatory framework leads to confusion about who regulates what, and whether some products are indeed subject to regulation, which leads to a loss of public confidence in biotechnology products. It benefits everyone to have a clear and coherent regulatory system: consumers and developers, and the American public.
